# An evaluation of 9-1-1 calls to assess the effectiveness of dispatch-assisted cardiopulmonary resuscitation (CPR) instructions: design and methodology

**DOI:** 10.1186/1471-227X-8-12

**Published:** 2008-11-05

**Authors:** Christian Vaillancourt, Manya L Charette, Ian G Stiell, George A Wells

**Affiliations:** 1 Ottawa Health Research Institute, Clinical Epidemiology Program, Ottawa, Canada; 2 Department of Emergency Medicine, University of Ottawa, Ottawa, Canada; 3 Department of Epidemiology and Community Medicine, University of Ottawa, Ottawa, Canada

## Abstract

**Background:**

Cardiac arrest is the leading cause of mortality in Canada, and the overall survival rate for out-of-hospital cardiac arrest rarely exceeds 5%. Bystander cardiopulmonary resuscitation (CPR) has been shown to increase survival for cardiac arrest victims. However, bystander CPR rates remain low in Canada, rarely exceeding 15%, despite various attempts to improve them. Dispatch-assisted CPR instructions have the potential to improve rates of bystander CPR and many Canadian urban communities now offer instructions to callers reporting a victim in cardiac arrest. Dispatch-assisted CPR instructions are recommended by the International Guidelines on Emergency Cardiovascular Care, but their ability to improve cardiac arrest survival remains unclear.

**Methods/Design:**

The overall goal of this study is to better understand the factors leading to successful dispatch-assisted CPR instructions and to ultimately save the lives of more cardiac arrest patients. The study will utilize a before-after, prospective cohort design to specifically: 1) Determine the ability of 9-1-1 dispatchers to correctly diagnose cardiac arrest; 2) Quantify the frequency and impact of perceived agonal breathing on cardiac arrest diagnosis; 3) Measure the frequency with which dispatch-assisted CPR instructions can be successfully completed; and 4) Measure the impact of dispatch-assisted CPR instructions on bystander CPR and survival rates.

The study will be conducted in 19 urban communities in Ontario, Canada. All 9-1-1 calls occurring in the study communities reporting out-of-hospital cardiac arrest in victims 16 years of age or older for which resuscitation was attempted will be eligible. Information will be obtained from 9-1-1 call recordings, paramedic patient care reports, base hospital records, fire medical records and hospital medical records. Victim, caller and system characteristics will be measured in the study communities before the introduction of dispatch-assisted CPR instructions (before group), during the introduction (run-in phase), and following the introduction (after group).

**Discussion:**

The study will obtain information essential to the development of clinical trials that will test a variety of educational approaches and delivery methods for telephone cardiopulmonary resuscitation instructions. This will be the first study in the world to clearly quantify the impact of dispatch-assisted CPR instructions on survival to hospital discharge for out-of-hospital cardiac arrest victims.

**Trial Registration:**

ClinicalTrials.gov NCT00664443

## Background

### Out-of-hospital cardiac arrest

Cardiac arrest refers to the sudden cessation of cardiac mechanical activity as confirmed by the absence of signs of circulation [1]. The victim collapses when the cardiac mechanical activity becomes too limited to provide adequate blood flow and oxygen to the brain and muscles. The victim is perceived to be lifeless if no vital signs are detectable (responsiveness, pulse, respirations). Electrical cardiac activity (ventricular fibrillation [VF], ventricular tachycardia [VT], or pulseless electrical activity [PEA]) seen on a cardiac monitor may become the only sign of vital activity. In the absence of cardiopulmonary resuscitation (CPR) and/or electrical defibrillation, such electrical cardiac activity is followed by asystole and then by death in a matter of minutes.

Cardiac arrest remains the leading cause of mortality in North America. The incidence of out-of-hospital cardiac arrest in Canada is estimated to be 55 per 100,000 of population [2], resulting in more than 17,875 deaths annually. Coronary artery disease is the most frequent condition leading to cardiac arrest [3]. More than 40% of all deaths from heart disease occur suddenly, and often constitute the victim's first manifestation of heart disease [4]. Sixty-five percent of all cardiac arrests occur outside the hospital setting [5], where the overall rate of survival to hospital discharge rarely exceeds 5% [2]. Survivors have a quality of life (Health Utilities Index Mark 3) similar to the general population [6]. Most cardiac arrest victims are men who are older than age 60, and still active members of society. They collapse in their own home 85% of the time and 50% are witnessed by a family member or bystander [2]. However, bystander CPR rates remain low in Canada, and rarely exceed 15% of all cases in Ontario [2].

### The Chain of Survival

The American Heart Association's "Chain of Survival" illustrates important concepts in the community response to out-of-hospital cardiac arrest [7]. The chain metaphor implies that cardiac arrest care is only as strong as its weakest link among the four links in the chain: 1) Early access: When dialling 9-1-1, a caller is put in communication with personnel that will appropriately dispatch police, fire, emergency medical services (EMS), or all three. In the case of a medical emergency, the call will rapidly be transferred to a medical dispatch centre. Dispatch Officers collect information on the nature of the medical emergency and dispatch the appropriate EMS unit(s), often as more information is being collected. 2) Early CPR: CPR can be defined as a succession of lung insufflations and chest compressions performed by a rescuer with the intention of restoring spontaneous circulation. Although police, fire, and EMS paramedics have all been trained to perform CPR in cases of cardiac arrest, it is what occurs during the first minutes before their arrival that is most crucial to the victim. A victim is almost four times more likely to survive a cardiac arrest event when receiving citizen bystander CPR before emergency personnel arrives [OR 3.7 (95% CI 2.5–5.4)] [8]. 3) Early defibrillation: Defibrillation occurs when myocardial cells in a chaotic or abnormal electrical rhythm, VF or VT, are depolarized at the same time by the delivery of an electrical current. This results in the re-establishment of a rhythmic and organized heart beat. Defibrillation can only occur when the heart exhibits disorganized electrical activity and is never successful in the case of asystole or PEA. 4) Early Advanced Care: Advanced cardiac life support care is defined by the use of definitive airway management such as endotracheal intubation, intravenous access and administration of drugs. Such drugs serve the purpose of increasing the coronary perfusion pressure by increasing peripheral vascular resistance (Epinephrine, Vasopressin), or to promote arrhythmia termination either alone by acting on myocardial cell electric action potential and/or by facilitating defibrillation (Lidocaine, Procainamide, Amiodarone). This being said, early advanced care failed to improve survival to hospital discharge in the largest prospective prehospital study conducted to date [8].

### Cardiac arrest location and public access defibrillation (PAD) programs

Following the publication of the Public Access Defibrillation (PAD) trial in 2004 [9], PAD programs received the highest endorsement from the American Heart Association. This study randomized community units to one of two emergency response systems consisting of either lay volunteers trained in CPR alone, or in CPR and the use of Automatic External Defibrillators (AED). Survival to hospital discharge from a cardiac arrest occurring in a public location increased as a result of the AED program [Relative Risk 2.0 (95% CI 1.07 to 3.77); P = 0.03] [9].

Unfortunately, since 85% of all cardiac arrests occur in a residential location [2], even the highest achievable survival rate in the remaining 15% of cardiac arrests occurring in a public location would have a limited impact on overall cardiac arrest survival. Stakeholders should consider the issues surrounding the implementation of a residential-access defibrillation program, and/or an alternative intervention most susceptible to improve bystander CPR rates in residential locations.

### Low bystander CPR rates

Data from Seattle indicates that a survival rate of 30% can be achieved for witnessed VF cardiac arrest victims receiving bystander CPR [10]. Other communities such as Akita and Otsu, Japan report overall survival rates from cardiac arrest of 15% and 9%, respectively, in association with bystander CPR rates of 49% and 29% [11]. In comparison, citizen bystander CPR and overall survival rates are rather modest in most Canadian provinces, rarely exceeding 15% and 5% respectively [2].

Various attempts have been made to improve bystander CPR rates in the past. Two very popular and contrasting approaches are mass CPR training events and targeted CPR training of family members of patients suffering from cardiovascular disease. The objective of the mass CPR training is to teach as many CPR providers as possible from the general population in a group setting. Although mass CPR training events can reach groups of a few hundred to thousands of participants at a time [12-15], these events usually attract young participants unlikely to witness cardiac arrest. In addition, they are not cost-effective [16] and their effect on survival has not been demonstrated. Targeting the population at large may not achieve the desired goal [17,18].

The second approach, targeted CPR training, involves spouses and other family members of cardiac arrest victims, the people most likely to witness the event [19-21]. Many authors have suggested targeting family members of patients with known cardiovascular disease for CPR training [22-33]. As few as 9% of this target group have generally received CPR training whereas the highest rate is found in Detroit at 47.4% [21,34-37]. Interest in CPR training appears to decrease with advancing age [38-42]. When a group of elders were asked why they had not sought CPR training in the past, most gave no specific reason, or mentioned the inconvenience of having to leave the house, bad health, or cost [43,44]. Some respondents did not understand why they should learn CPR when they can call 9-1-1 [45]. On the other hand, CPR training was shown to decrease anxiety, and increase emotional adjustment in family members of cardiac arrest survivors [46-48]. Many victims of cardiac arrest never had any documented cardiovascular disease preceding the event; therefore, targeting family members of patients with known cardiovascular disease may not achieve the desired goal either.

A systematic review of the literature on bystander CPR has recently been published [49]. Based on an extensive review of the evidence available at the time, the authors indicated that there is support (in the form of at least one good quality randomized controlled trial) for the following as potential ways of increasing low bystander CPR rates: 1) 9-1-1 dispatch-assisted CPR instructions, 2) teaching CPR to family members of cardiac patients, 3) using self training videos to learn CPR, 4) maximizing the time spent using manikins during CPR training, and 5) teaching the concepts of ambiguity and diffusion of responsibility. Mass CPR training events were not supported by the literature as a way of increasing low bystander CPR rates.

Irrespective of the teaching method, retention of CPR knowledge and skills is poor [50-52]. The Heart and Stroke Foundation of Canada currently recommends yearly retraining [53]. There is a large amount of literature demonstrating a significant decrease in CPR knowledge and skills after one year [54-66]. Trainees may go back to pre-training levels as early as six months after their CPR class [67-71]. Some evidence exists that re-training may be protective against a decline in CPR skills [72].

It appears that dispatch-assisted CPR instructions could represent the best CPR training alternative thus far. This intervention combines the benefits of training a large number of citizens with the highly targeted approach of providing CPR teaching or reminders to 9-1-1 callers reporting a victim in cardiac arrest. In addition, it partially circumvents the issue of knowledge and skill decay with time.

### Dispatch-assisted CPR instructions

In Canada, once the presence of a medical emergency has been established, 9-1-1 callers are rapidly put in communication with a medical dispatch centre. Dispatch Officers working at the medical dispatch centre can be a paramedic, a nurse, or a non-health care professional. Many medical dispatch centres in Canada, however, employ dispatchers with no professional health care background. The dispatchers receive six weeks of training with an instructor in order to learn how to navigate a set of dispatch instructions and this is followed by a six-month preceptorship. Dispatch Officers must recognize and process a large number of medical emergencies. They follow algorithms as part of a "dispatch protocol". Each question asked by the Dispatch Officer of the 9-1-1 caller is standardized such that a given set of instructions will be initiated for a specific medical emergency. For cardiac arrest, Dispatch Officers initiate CPR instructions over the telephone while emergency response vehicles are on their way to the location.

There are several types of "dispatch protocols" available on the market. Dr. Jeff Clawson and the National Academies of Emergency Dispatch have developed the Medical Priority Dispatch System (MPDS). It is used in most North American cities and in some European communities. Ontario uses a different dispatch system developed by the Ministry of Health and Long-Term Care. Niagara Falls and Toronto are the only communities using the MPDS in Ontario. With regard to dispatch-assisted CPR instructions, there is only one major difference between the two systems – chest compressions and mouth-to-mouth are still being taught by Ontario Dispatch Officers whereas the MPDS teaches chest compressions only.

### Agonal breathing and the ability to recognize cardiac arrest

In the first minutes following cardiac arrest, some victims will take short, laboured, noisy gasping breaths. This abnormal breathing, otherwise known as agonal breathing, could be present in as many as 30% of all cardiac arrest victims, but a large observational study is needed to confirm these numbers. Agonal breathing may limit the ability of Dispatch Officers to make a diagnosis of cardiac arrest [73,74]. Some evidence suggests that agonal breathing can be misinterpreted as a sign of life, resulting in the inappropriate withholding of dispatch-assisted CPR instructions. The ability of Dispatch Officers to accurately detect cardiac arrest over the telephone ranges between 68% and 90% in some studies [75,76].

In contrast, in a review of the Seattle experience, Dispatch Officers initiated CPR instructions after erroneously making the diagnosis of cardiac arrest over the telephone 14% of the time [77]. For various reasons, since telephone CPR instructions do not always result in CPR being performed on the victim, only 4.3% of victims believed to be in cardiac arrest erroneously received CPR [77]. This is consistent with the 2005 International Consensus Guidelines on CPR in that, when in doubt about the presence or absence of signs of life, it is recommended to err on the safe side and initiate CPR. No adverse events were reported in the small group receiving CPR erroneously.

### Ability of 9-1-1 callers to follow dispatch-assisted CPR instructions

Two studies of recorded 9-1-1 calls determined that CPR instructions were appropriate and possible in only 30% to 37% of all confirmed cardiac arrest cases [78,79]. While a majority of callers were emotionally capable of following instructions [75,80,81], the telephone was not in close range of the victim as often as 50% of the time [80-82]. Many of these studies are now more than 10 years old; with the introduction of cell phones and cordless phones, the ability of providing CPR instructions over the telephone likely has improved significantly.

It also appears that ventilation instructions over the telephone can be relatively time consuming. In a randomized, controlled study by Hallstrom, it took an additional 1.4 minutes to complete the mouth-to-mouth instructions compared to doing immediate chest compressions without ventilations [83]. In some cases, this additional delay can result in the paramedics arriving before the chest compressions are initiated. Although results from the Hallstrom study did not reach statistical significance, survival to hospital discharge appeared to favour the chest-compression only group compared to traditional CPR with ventilation instructions (14.6% vs. 10.4%; P = 0.18) [84]. Currently, most 9-1-1 dispatch centers no longer offer ventilation instructions to callers reporting a witnessed cardiac arrest victim whose collapse occurred less than 10 minutes prior to the call.

### Pilot evaluation of dispatch-assisted CPR instructions in Ottawa, Canada

We recently conducted a pilot observational cohort study evaluating the frequency of agonal breathing during cardiac arrest, its impact on the ability of Dispatch Officers to recognize cardiac arrest, and the impact of dispatch-assisted CPR instructions on bystander CPR rates in Ottawa, Canada [85].

Audio recordings of all out-of-hospital cardiac arrest cases over two successive nine-month periods were obtained. The nine-month periods corresponded to the periods before and after the introduction of dispatch-assisted CPR instructions. There were 529 cardiac arrest cases during the two periods between July 1^st^, 2003 and December 31^st^, 2004 [85]. Victim characteristics were similar in the before (N = 295) and after (N = 234) phase: mean age was 68.3, 66.7% were male, collapse was witnessed 50.1% of the time, the mean time interval between 9-1-1 call to vehicle stop at the scene was 6:37 min:sec, VF/VT was present in 29.9%, and survival to hospital discharge was 4.0%. We located 82.1% of 9-1-1 recordings for the after period. Callers were female 63.5% of the time, the victim's spouses 29.2% of the time, and previously trained in CPR 24.0% of the time. Dispatch Officers recognized 56.0% (95% CI 48.9–63.0%) of cardiac arrest cases; agonal breathing was present in 37.0% (95% CI 30.1–43.9%) of all cardiac arrest cases and accounted for 50.0% (95% CI 39.1–60.9%) of missed diagnoses. CPR instructions were offered to 75.9% and accepted by 53.7% of callers; 26.3% declined instructions because of prior CPR training. 17.2% and 8.3% of callers provided ventilations and chest compressions as a result of the intervention. The longest delays occurred between call-to-cardiac arrest diagnosis (2:38), and during ventilation instructions (2:05). Bystander CPR rates increased from 16.7% to 26.4% (AR 9.7%; 95% CI 8.5–11.3% p = 0.006).

Before the introduction of dispatch-assisted CPR instructions, no other intervention had succeeded in improving bystander CPR rates in the community of Ottawa, Canada. The pilot project allowed the authors to determine the feasibility of collecting information of interest. The next logical step is to conduct a large, multi-centre trial in order to best evaluate the impact of dispatch-assisted CPR instructions.

## Objectives

The overall goal of this study is to better understand the factors leading to successful dispatch-assisted CPR instructions. This is essential in order to intervene to improve the effectiveness of these instructions, to further improve bystander CPR rates, and to save more victims of cardiac arrest. Specific objectives are to:

1) Determine the ability of Dispatch Officers to correctly diagnose cardiac arrest over the telephone;

2) Quantify the frequency and impact of perceived agonal breathing on cardiac arrest diagnosis by Dispatch Officers;

3) Measure the frequency with which dispatch-assisted CPR instructions can be successfully completed in out-of-hospital cardiac arrest cases; and

4) Measure the impact of dispatch-assisted CPR instructions on bystander CPR rates and survival for out-of-hospital cardiac arrest.

## Methods

### Study design and setting

**Figure 1:**
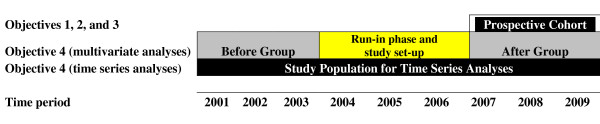
Study design and proposed statistical analyses.

This study will combine a before-after design with a prospective cohort design (Figure 1). Objective 4 (bystander CPR and survival rates) will be addressed using a before-after design, the "before" phase occurring prior to 2004, and the prospective "after" phase starting in 2007 (after the run-in period). Objectives 1, 2, and 3 will be addressed with a prospective cohort starting in 2007. The prospective cohort group and the "after" group will be the same group.

The study will take place in 19 large and small cities located in Ontario, Canada affiliated with 11 base hospital programs. The combined population in those 19 communities is 2,903,887 and ranges between 15,605 and 774,072 (2001 Statistics Canada census). Approximately 1,703 patients suffer cardiac arrest each year and receive treatment in these communities. All communities have sophisticated EMS services with first responding fire fighters and second-tier primary care (BLS-D) and advanced care (ALS) paramedics. All centres have been collecting high-quality data on system and cardiac arrest victim characteristics since 1994, and all have initiated dispatch-assisted CPR instructions between 2004 and 2005.

### Study population

All patients with out-of-hospital cardiac arrest (absence of a detectable pulse, unresponsiveness and apnea) meeting the following criteria will be enrolled in the study:

a) presumed cardiac origin;

b) occurring in an eligible study community; and

c) for which resuscitation is attempted by a bystander and/or the emergency responders; or for which dispatch-assisted CPR instructions are being provided.

Case definitions will follow the Utstein Style guidelines for reporting cardiac arrest data [1].

Patients meeting any of the following criteria will be excluded:

a) patients younger than 16 years (cardiac arrest usually respiratory and rare in this population);

b) patients who are "obviously dead" as defined by the Ambulance Act of Ontario (decomposition, rigor mortis, decapitation, or other);

c) trauma victims, including hanging and burns; or

d) patients with cardiac arrests clearly of other non-cardiac origin including drug overdose, carbon monoxide poisoning, drowning, exsanguination, electrocution, asphyxia, hypoxia related to respiratory disease, cerebrovascular accident, and documented terminal illness.

### Method of assessment and data collection

#### Diagnosis of Cardiac Arrest by Dispatch Officers

According to protocols, Dispatch Officers assume the presence of cardiac arrest when the 9-1-1 caller describes a victim that is lifeless (victim is unresponsive, not breathing, or not breathing normally). Dispatch Officers will notify the medical dispatch centre after every occasion where assisted CPR instructions were initiated. Using a unique identifier, these interventions/cases will later be cross-referenced with the regional cardiac arrest registry for measuring diagnostic accuracy. In addition, a research assistant will later listen to the recordings of each cardiac arrest intervention and document on a standardized and piloted data collection form the reasons why cardiac arrest may not have been recognized by the Dispatch Officer.

#### Diagnosis of Cardiac Arrest of Cardiac Origin

In order to verify the diagnostic accuracy of Dispatch Officers, the status of a cardiac arrest victim will be confirmed using the Utstein definition for cardiac arrest of cardiac origin [1].

Cardiac arrest will first be documented at the scene by the paramedic crew, and subsequently confirmed by the base hospital physician or coroner. Cardiac arrest will be defined as per the Utstein criteria for reporting data on cardiac arrest research.

#### Agonal breathing

Can be defined as abnormal breathing, gurgling sound, moaning, or otherwise. Dispatchers Officers are required by protocol to inquire about the presence or absence of agonal or abnormal breathing. A research assistant at each base hospital will systematically review the recordings of all cardiac arrest calls, and use a piloted data collection form to independently document the presence or absence of agonal breathing, as described by the 9-1-1 caller to the Dispatch Officer. Consistency in recognizing agonal breathing among research assistants will be assured with the use of training 9-1-1 communication material before the study starts.

#### Successful Completion of Dispatch-Assisted CPR Instructions

is defined as CPR instructions provided by a Dispatch Officer to a 9-1-1 caller reporting a cardiac arrest victim, resulting in the administration of chest compressions to that victim by a bystander before EMS arrival. Once again, a research assistant will systematically review the recordings of all cardiac arrest calls using a standardized and piloted data collection form in order to collect information on variables pertaining to the successful delivery of dispatch-assisted CPR instructions. Paramedics are required by protocol to document if bystander CPR was ongoing before their arrival.

#### Bystander CPR

"Bystander" CPR is performed by a person who is not part of an organized emergency medical system. The presence or absence of ongoing bystander CPR will be assessed by the first member of the emergency response team to arrive at scene, and will be documented.

#### Survival to Hospital Discharge

is defined as survival of a cardiac arrest victim to hospital discharge. Survival to hospital discharge will be assessed via hospital medical records or via the coroner's office.

### Data analysis

#### Cardiac Arrest Recognition

Sensitivity, specificity and receiver operating characteristic (ROC) curves will be computed.

#### Agonal Breathing

Descriptive statistics with 95% confidence intervals (95%CI), and kappa statistics with 95% CI will be calculated to measure the level of agreement between the dispatcher and the research assistant during the study, and among research assistants using training materials before the study.

#### Dispatch-Assisted CPR Instructions

Descriptive statistics with 95% CIs will be calculated to describe successes in various stages of the implementation of CPR instructions, to describe the caller's receptiveness of the instructions, and various time delays occurring during dispatch-assisted CPR instructions.

#### Bystander CPR and Survival Rates

Descriptive statistics with 95% CIs will be calculated for bystander CPR and survival rates. In addition, two statistical modelling approaches will be considered: 1) A univariate and a stepwise logistic regression analysis to control for potential confounding effects of variables possibly associated with bystander CPR and survival rates. These variables include age, gender, initial cardiac rhythm, and EMS response time intervals. Bystander CPR rates and cardiac arrest survival rates in the "before" phase (prior to 2004) will be compared to the "after" phase (after the run-in period finishing in 2006) controlling for these confounding variables. 2) A time series analysis to analyse trends (quarterly) over the whole study period (including the run-in phase), and identify a possible point of inflection due to the implementation of dispatch-assisted CPR instructions. This will determine if bystander CPR and survival rate inflections are significant, controlling for trends over time. This analysis will be performed for each community and then the results may be combined in a meta-analysis after assessing homogeneity of the results across the communities.

### Sample size

The main objective of this study is to measure the effect of dispatch-assisted CPR instructions on survival to hospital discharge for out-of-hospital cardiac arrest victims. The required sample size has been estimated based on a dichotomous response variable from two independent samples (before and after the introduction of dispatch-assisted CPR instructions). The required sample size is based on an expected survival rate of 5% in the before group (based on previous studies conducted in the study communities [8]), an expected survival rate of 7% in the after group (based on mathematical modelling and scientific reports), an average survival rate of 6% for all study participants and a 2% minimal clinically important difference in survival rate. Using these figures and setting power at 90%, 2,962 cardiac arrest victims will be required for each group.

### Ethics approval

Research ethics board approval was obtained from The Ottawa Hospital (protocol #2007233-01H). As the study will not affect usual practice, there were no specific ethical concerns. Informed consent is not required because it is impossible to obtain in emergency situations such as cardiac arrest, and because patients will not be randomized to receive different therapies. No identifying information on either the dispatcher or the patient will be recorded.

## Discussion

Dispatch-assisted CPR instructions are relatively new in the field of cardiac arrest resuscitation. Their delivery methods are based on limited scientific evidence, and need to be further optimized. This study will provide essential information on modifiable factors to improve the quality of dispatch-assisted CPR instructions, further improve bystander CPR rates, and save more cardiac arrest victims. First, we will identify modifiable factors that could increase the current cardiac arrest diagnostic accuracy of Dispatch Officers. Second, we will quantify the impact of agonal breathing on the Dispatch Officer's diagnostic accuracy in order to develop educational strategies designed to help them recognize this condition. Third, we will identify modifiable factors influencing the ability of 9-1-1 callers to comply with CPR instructions provided over the telephone. Finally, we will convincingly determine the impact of dispatch-assisted CPR instructions on bystander CPR and survival rates.

This information is essential to develop clinical trials testing a variety of educational approaches and delivery methods for telephone CPR instructions. This will be the first study worldwide to clearly quantify the impact of dispatch-assisted CPR instructions on survival to hospital discharge for out-of-hospital cardiac arrest victims. We anticipate our study will show that dispatch-assisted CPR instructions contributed to saving as many as 40 lives per year in the participating centres alone, and as much as 360 lives annually in Canada.

## Competing interests

The authors declare that they have no competing interests.

## Authors' contributions

CV conceived of the study and drafted the manuscript. MC helped to draft and edit the manuscript. GW assisted with the statistical design and methodology. IS assisted with the methodology, revised it critically for important intellectual content, and helped to draft the manuscript. All authors read and approved the final manuscript.

## Pre-publication history

The pre-publication history for this paper can be accessed here:


